# Death of Dr. Amos Twitchell

**Published:** 1850-07

**Authors:** 


We regret to learn from the Boston Medical and Surgical Journal, the death of Dr. Amos Twitchell. He died at Keene, New Hampshire, on the 26th of May, aged 60 years. Dr. Twitchell was known as one of the first physicians and surgeons of New England. He was a bold and skilful operator, and greatly beloved, as well for his professional attainments as his high social qualities.

---

The University of Pennsylvania has determined to adhere to the six months course.

---

Dr. G. B. Wood has gone to Europe to collect materials illustrative of his lectures on the Practice of Medicine in the University of Pennsylvania.

